# Physical activity intensity, bout-duration, and cardiometabolic risk markers in children and adolescents

**DOI:** 10.1038/s41366-018-0152-8

**Published:** 2018-07-13

**Authors:** Jakob Tarp, Abbey Child, Tom White, Kate Westgate, Anna Bugge, Anders Grøntved, Niels Wedderkopp, Lars B. Andersen, Greet Cardon, Rachel Davey, Kathleen F Janz, Susi Kriemler, Kate Northstone, Angie S. Page, Jardena J. Puder, John J. Reilly, Luis B. Sardinha, Esther M. F. van Sluijs, Ulf Ekelund, Katrien Wijndaele, Søren Brage, On behalf of the International Children’s Accelerometry Database (ICAD) Collaborators

**Affiliations:** 1 Research Unit for Exercise Epidemiology, Department of Sports Science and Clinical Biomechanics, Centre of Research in Childhood Health, University of Southern Denmark, Odense, Denmark; 2 Medical Research Council Epidemiology Unit, University of Cambridge, Cambridge, UK; 3 University of Cambridge, Cambridge, UK; 4 Sports Medicine Clinic, The Orthopedic Department, Hospital of Lillebaelt Middelfart, Institute of Regional Health Research, University of Southern Denmark, Odense, Denmark; 5 Department of Teacher Education and Sport, Western Norwegian University of Applied Sciences, Sogndal, Norway; 6 Department of Movement and Sports Sciences, Ghent University, 9000 Ghent, Belgium; 7 Centre for Research and Action in Public Health, University of Canberra, Canberra, Australia; 8 Department of Health and Human Physiology, University of Iowa, Iowa City, USA; 9 Epidemiology, Biostatistics and Prevention Institute, University of Zurich, Zurich, Switzerland; 10 Bristol Medical School, University of Bristol, Bristol, UK; 11 Centre for Exercise, Nutrition and Health Sciences, School for Policy Studies, University of Bristol, Bristol, UK; 12 Service of Endocrinology, Diabetes and Metabolism and Division of Pediatric Endocrinology, Diabetes and Obesity, University Hospital Lausanne, Lausanne, Switzerland; 13 University of Strathclyde, Physical Activity for Health Group, School of Psychological Sciences and Health, Glasgow, Scotland UK; 14 Exercise and Health Laboratory, Faculty of Human Kinetics, Universidade de Lisboa, Lisbon, Portugal; 15 Centre for Diet and Activity Research (CEDAR), University of Cambridge, Cambridge, UK; 16 Department of Sports Medicine, Norwegian School of Sport Sciences, Oslo, Norway

## Abstract

**Objectives:**

To determine the role of physical activity intensity and bout-duration in modulating associations between physical activity and cardiometabolic risk markers.

**Methods:**

A cross-sectional study using the International Children’s Accelerometry Database (ICAD) including 38,306 observations (in 29,734 individuals aged 4–18 years). Accelerometry data was summarized as time accumulated in 16 combinations of intensity thresholds (≥500 to ≥3000 counts/min) and bout-durations (≥1 to ≥10 min). Outcomes were body mass index (BMI, kg/m^2^), waist circumference, biochemical markers, blood pressure, and a composite score of these metabolic markers. A second composite score excluded the adiposity component. Linear mixed models were applied to elucidate the associations and expressed per 10 min difference in daily activity above the intensity/bout-duration combination. Estimates (and variance) from each of the 16 combinations of intensity and bout-duration examined in the linear mixed models were analyzed in meta-regression to investigate trends in the association.

**Results:**

Each 10 min positive difference in physical activity was significantly and inversely associated with the risk factors irrespective of the combination of intensity and bout-duration. In meta-regression, each 1000 counts/min increase in intensity threshold was associated with a −0.027 (95% CI: −0.039 to −0.014) standard deviations lower composite risk score, and a −0.064 (95% CI: −0.09 to −0.038) kg/m^2^ lower BMI. Conversely, meta-regression suggested bout-duration was not significantly associated with effect-sizes (per 1 min increase in bout-duration: −0.002 (95% CI: −0.005 to 0.0005) standard deviations for the composite risk score, and −0.005 (95% CI: −0.012 to 0.002) kg/m^2^ for BMI).

**Conclusions:**

Time spent at higher intensity physical activity was the main determinant of variation in cardiometabolic risk factors, not bout-duration. Greater magnitude of associations was consistently observed with higher intensities. These results suggest that, in children and adolescents, physical activity, preferably at higher intensities, of any bout-duration should be promoted.

## Introduction

Cardiovascular disease accounted for 17.6 million deaths worldwide in 2016, making it the leading cause of non-communicable disease mortality [1]. While the disease is generally a concern in adulthood, cardiometabolic risk factors may be present from a much earlier age, for example endothelial damage that leads to atherosclerosis can develop during adolescence [2]. In addition, previous evidence suggests cardiometabolic risk factors may track from childhood and adolescence into adulthood [3]. This makes it important to understand the modifiable determinants of cardiometabolic risk factors in young people. One such determinant is participation in physical activity [4–6].

Current national and international physical activity guidelines recommend adults should accumulate moderate-to-vigorous physical activity (MVPA) or vigorous physical activity in bouts of at least 10 min duration [7, 8]. For children and adolescents, a daily total of at least 60 min of MVPA is recommended [7, 8] but many countries (including the U.S. [9], U.K. [7], Australia [10], and Canada [11]) do not specify any minimum bout-duration for MVPA. However, a minimum bout-duration of 5 min was included in a previous version of the Canadian guidelines [12] and is included in some national guidelines [13]. Providing the optimal guidance on how to perform health-enhancing physical activity is important for authorities and clinicians. However, whether short bouts of activity confer similar benefits to longer durations remains unclear and available evidence on this issue remains scarce in young people [14–17]. Accelerometry is currently the de facto standard of objective physical activity assessment in large-scale epidemiological studies [18]. It is well-established that accelerometry-determined MVPA levels are highly influenced by the choice of intensity threshold [19] but it has not been sufficiently explored how varying the intensity threshold impacts on associations with cardiometabolic risk factors. Further, whether higher (or lower) intensity physical activity may be particularly beneficial for cardiometabolic risk factors at longer bout-durations has yet to be examined. Therefore, the purpose of this study was to assess how physical activity of different intensities and accumulated in bouts of varying duration relates to cardiometabolic health in young people. Since bouted activity is highly correlated with total activity [14, 17], we also examined if an additional benefit of longer duration activity was evident after accounting for variation in total physical activity.

## Methods

### Study design and participants

This study was based on secondary data from the International Children’s Physical Activity Database (ICAD, http://www.mrc-epid.cam.ac.uk/research/studies/icad/) which contains harmonized objectively measured physical activity data from studies in youth across the world [20]. All studies were based on participant/parental written informed consent and consulted with their respective research boards to ensure appropriate ethical approval of data-sharing. Included studies were conducted between 1997 and 2009 in 11 countries [21–38]. A total of 44,869 physical activity files were available from the ICAD database. Participants with sufficient physical activity data (criteria given below) and data on any of the considered outcomes were eligible for this study. After exclusion of participants due to insufficient (detailed below) or unreliable data (flagged by ICAD central processing) [20]—physical activity data (*n* = 5861), age outside the 4–18 year range (*n* = 370), or missing outcome data (*n* = 332)—a final sample size of 38,306 observations from 29,800 unique individuals was included. Two or more observations were available from 25.5% of the included sample.

### Physical activity data reduction

A detailed description of the protocol for harmonization of physical activity data is provided elsewhere [20]. In short, available raw data files were reanalyzed to create directly comparable variables across all contributing ICAD studies. Epoch length was harmonized to 60 s due to the lack of availability of shorter epochs in older studies (KineSoft version 3.3.20, KineSoft, Saskatchewan, Canada). For this analysis, all epochs producing counts ≥30,000 were deemed incompatible with human movement behavior and considered non-wear. Non-wear was further defined as strings of identical count values for >60 consecutive min in the data time-series (https://github.com/Thomite/pampro.git). These strings were removed before summation of activity and wear time. As strings of identical count values are unlikely to represent true movement behavior, this approach will both remove continuous zero counts and reduce data with technical malfunction (i.e., count plateau). To avoid extreme outliers, days with recorded mean counts/min above the 99.9th percentile (2125 counts/min (cpm)) or below the 0.1th percentile (36 cpm) were discarded. Three or more days of ≥500 mins of wear-time between 7 a.m. and midnight (data outside these hours was discarded) were required for a participant to be included in this analysis [18]. To investigate the effect of higher intensity of physical activity we defined four increasing, but arbitrarily chosen, intensity thresholds; ≥500 cpm, ≥1000 cpm, ≥2000 cpm, and ≥3000 cpm. Further, we summarized time above these intensity thresholds as uninterrupted bouts of ≥1 (includes all activity), ≥2, ≥5 (medium), and ≥10 (long) min. A bout was terminated when counts dropped below the respective intensity threshold. As an example, the following min-by-min accelerometer sequence 0-3000-3000-3000-3000-3000-500-500-3000-3000-3000-3000-3000-0 would therefore be summarized as 5 + 5 = 10 min spent in ≥5 min bouts ≥3000 counts/min but zero min spent in ≥10-min bouts (and similar for ≥2000 and ≥1000 counts/min intensities), whereas there would be 12 min accumulated in all the ≥500 counts/min bout variables. Variables were derived for each day separately and averaged across valid days for analysis. These data reductions lead to 16 combinations of intensity and bout-duration.

### Assessment of cardiometabolic risk factors

Outcome variables consisted of two anthropometric (waist circumference and body mass index (BMI)) and five biological (insulin, glucose, triglyceride, HDL-cholesterol, and mean arterial pressure (MAP, calculated as 1/3 × systolic blood pressure + 2/3 × diastolic blood pressure) [39]) markers reflecting established cardiometabolic risk factors. Standardized methods were used to measure height and weight across all studies with BMI calculated as weight (in kilograms) divided by height (in meters) squared. BMI was used to define overweight and obesity (World Obesity Federation cut-offs) [40]. Waist circumference was measured by the same procedure (WHO) in all contributing studies except the U.S. National Health and Nutrition Examination Survey (NHANES). The latter used a metal anthropometric tape placed at the midaxillary line (just above the iliac crest) [33, 34], as opposed to the midpoint between the lowest rib and iliac crest [21, 22, 27, 28, 31, 32, 35–37]. We converted NHANES data to WHO measurement methodology by applying a correction formula [41]. Blood pressure was measured in 10 studies, all using repeated measurements with automated [21, 27, 28, 32, 36] or manual [33, 34] methods after at least 5 min of rest. Eight studies obtained fasting measures of lipid metabolism (triglyceride and HDL-cholesterol) and 7 studies measured glycaemic metabolism in the basal state (fasting glucose and insulin). All used standardized procedures [27, 28, 31, 33, 34]. To maximize information on the latent cardiometabolic risk profile, we additionally calculated two composite risk scores using standardized values (*z*-scores) of the risk factors [42]. The first composite score included BMI, the homeostasis assessment model of insulin resistance (HOMA-IR) [43], triglyceride, MAP, and inverse HDL-cholesterol. The second composite score was identical but excluded BMI (non-adiposity composite score). All variables were standardized for age and sex with MAP additionally standardized for body-height. BMI, HOMA-IR, and triglyceride were log-transformed before standardization. The composite score was standardized to a mean of zero and standard deviation of one before analysis.

### Statistical analysis

Central tendencies of continuous variables are presented as mean (standard deviation) or median (25th–75th percentiles) based on distributional properties. Bout/intensity inter-correlations were explored using Spearman’s partial correlation controlling for age, sex, wear-time, and study. Data from studies was pooled into one dataset, and separate multivariable linear mixed-effects regression models were used to analyze associations between the 9 outcomes and 16 combinations of intensity and bout durations while including the co-variates age, sex, and wear-time. Body-height was additionally included when MAP and waist-circumference were outcomes. A mixed-effects logistic regression model was used to calculate odds of being overweight/obese. The non-adiposity composite score, insulin, glucose, triglycerides, HDL-cholesterol, and MAP were additionally controlled for BMI in secondary models. Post-hoc models including age-by-intensity/bout-duration and weight status (normal weight versus overweight/obese)-by-intensity/bout-duration interaction terms were constructed to examine potential heterogeneity in associations. In all models, individual participants and studies were modeled as “random-effects” except in the logistic model where only one observation per individual was included due to failure of the models to converge (the earliest observation was used). Additional adjustment for number of included days produced minimal changes in coefficients. Regression models were visually inspected for normal-distribution of residuals, variance homoscedasticity, and linearity between independent and dependent variables, as well as for influential observations (Cook’s D). All model assumptions were verified and no transformation of variables was necessary. Regression coefficients and 95% confidence intervals (CIs) are presented graphically in the form of forest plots, and represent the difference in outcome per 10 min/day positive difference in physical activity. To directly model whether physical activity spent in medium or long bouts confers an additional health benefit over an identical amount of time spent in shorter bouts of physical activity, we used an isotemporal substitution approach [44]. These models took the form (omitting error term):

Y = β_0_ + β_1_Physical Activity_≥5-9 min bouts at intensity _+ β_2_Physical Activity_≥10 min bouts at intensity_ + β_3_Total Physical Activity_at intensity_ + β_4_Wear-time + β_5_Age + β_6_Sex + ζ_1_Study + ζ_2_Participant

This model constrains total physical activity above the intensity threshold, thereby allowing for investigation of its composition [45]. The coefficients β_1_ and β_2_ thus represents the effect of substituting time spent in physical activity of 1–4 min duration (short bout-duration) with an equal amount of time spent in medium or long bout-durations of MVPA [44]. We explored linear trends in the influence of intensity and bout-duration on the outcomes by including estimates from mixed linear regressions in a meta-regression model [46]. CI’s in meta-regression models were adapted to account for non-independence of coefficients [47] by recalculating the standard error as: (√(number of coefficients (20)–1)) × the standard error obtained from the meta-regression. An intensity-by-bout duration interaction term was added in a separate meta-regression model to explore potential heterogeneity in associations across bout duration/intensity combinations. Estimates for bout-durations of ≥3 and ≥7 min (with intensity thresholds at ≥500 cpm, ≥1000 cpm, ≥2000 cpm, and ≥3000 cpm) were added to the meta-regression to increase information about the shape of the associations. Analyses were conducted using Stata/IC version 15.0. Significance tests were two-sided, and *p* values less than 0.05 were considered statistically significant. We did not include adjustment for multiple testing and provide an interpretation of data based on the pattern of results.

## Results

### Characteristics of the study sample

**Fig. 1:**
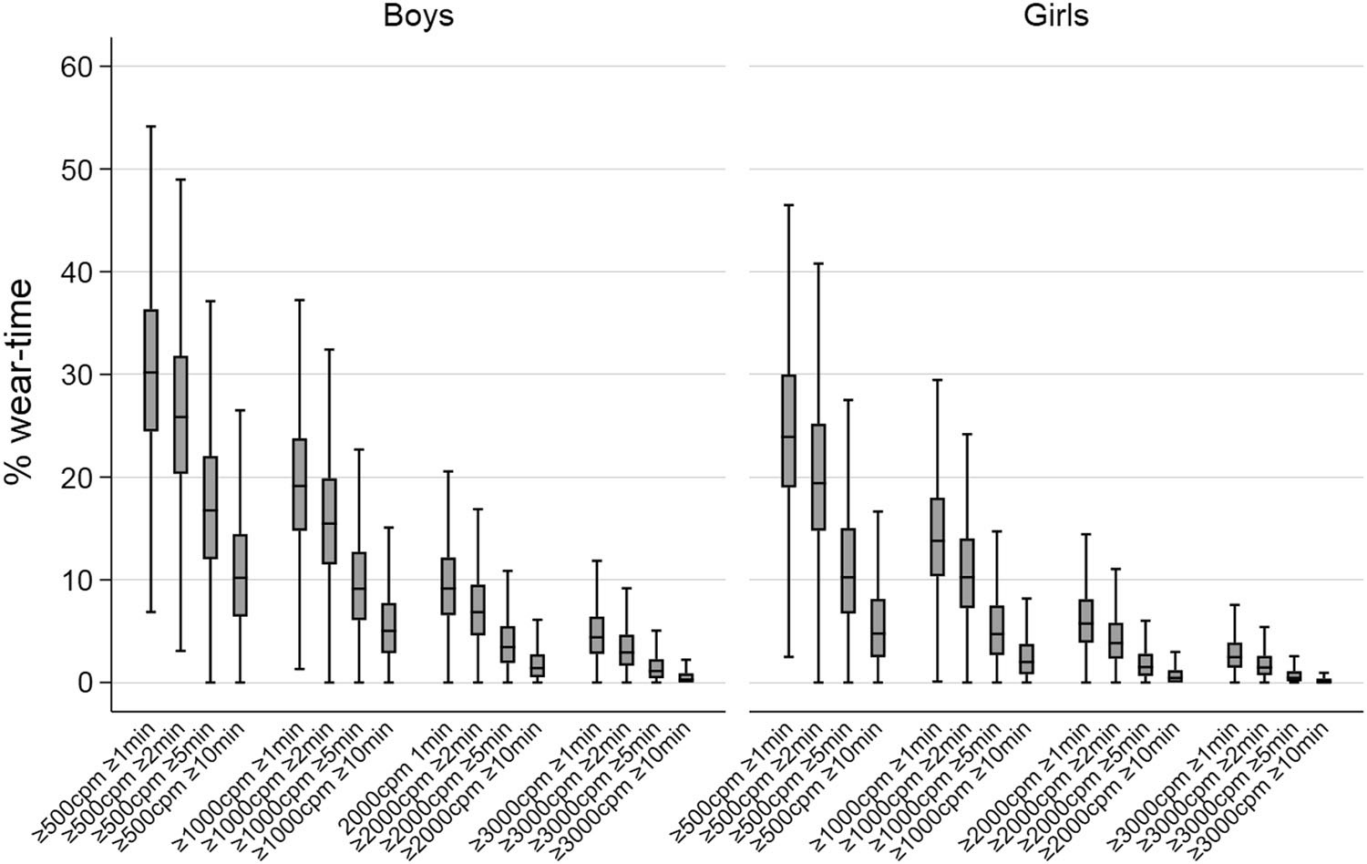
Activity patterns in 10,990 boys and 18,810 girls. Box-plot displays percentage of wear-time spent in intensity/bout combinations in boys and girls. Outside values not shown

Participant and study characteristics, including number of available studies and participants, are presented in Table 1, Table 2 and in Supplementary File Table S1 and Fig. S1. The median age of participants was 11.7 (11.1–13.6) years and 26% of the sample was overweight or obese. Each observation contributed a median of 6 (4–6) days with a mean of 13.2 (1.2) hours of wear-time/day. The median percentage of wear-time ≥500 counts/min, ≥1000 counts/min, ≥2000 counts/min, and ≥3000 counts/min were 26.3% (20.6–32.9%), 15.7% (11.6–20.7%), 6.9% (4.6%–9.8%), and 3.1% (1.8–4.9%), respectively. Boys spent a higher percentage of their time above each intensity/bout combination threshold (all *p*-values <0.001, Fig. 1). Correlations between bout-durations were high but decreased with higher intensity thresholds (correlation matrix shown in Supplementary File Table S2).

**Table 1:** Participant characteristics

	Girls (*n* = 18,810)^a^	Boys (*n* = 10,990)^a^
Age (years)	11.8 (10.6 – 13.8)	11.4 (9.6 – 12.0)
Body-height (cm)	152.4 (141.9 – 159.8)	145.7 (135.5 – 155.6)
Body-weight (kg)	45.6 (35 – 55.7)	38.0 (30.2 – 49.8)
Wear-time (h/day)^b^	13.2 (1.3)	13.1 (1.3)
Counts/min	453 (348 – 586)	620 (491 – 768)

Values are median with 25th–75th percentile unless noted otherwise

^a^Unique individual participants

^b^Mean (SD)

**Table 2:** Outcome characteristics

	No. of studies	Girls (%)	*n* ^a^	Median^b^	25th–75th percentile^b^	ICC study	ICC participant	Contributing studies
Composite risk score	6	52	4279	−0.05	−0.65 to 0.61	0.08	0.51	4, 5, 6, 10, 12, 15
Non-adiposity composite score	6	52	4279	−0.01	−0.66 to 0.66	0.08	0.35	4, 5, 6, 10, 12, 15
Insulin (pmol/l)	7	52	4649	42.78	27.28 to 64.80	0.07	0.65	4, 5, 6, 10, 12, 15, 20
Glucose (mmol/l)	7	52	4685	5	4.70 to 5.30	0.17	0.13	4, 5, 6, 10, 12, 15, 20
Triglyceride (mmol/l)	8	52	5027	0.69	0.51 to 0.95	0.08	0.51	4, 5, 6, 10, 11, 12, 15, 20
Mean arterial pressure (mmHg)	10	52	13 598	73.8	69.2 to 78.9	0.16	0.35	1, 4, 5, 6, 9, 10, 11, 12, 14, 15
HDL-c (mmol/l)	8	51	7386	1.43	1.22 to 1.68	0.03	0.55	4, 5, 6, 10, 11, 12, 15, 20
BMI (kg/m^2^)	21	62	29 734	18.7	16.5 to 21.8	0.05	0.86	all
Waist circumference (cm)	14	52	18 992	64.5	58.8 to 72.0	0.07	0.80	1, 4, 5, 6, 9, 10, 11, 12, 13,
Overweight/obese (%)	21	62	29 734	18/8				all

^a^Unique participants

^b^For prospective studies, only study “baseline” data is included in the table. Overweight and obesity defined according to World Obesity Federation cut-offs. Study indicators (NHANES waves counted separately): 1: ALSPAC, 2: Belgium Pre-School Study, 3: CLAN, 4: CoSCIS, 5: Danish EYHS, 6: Estonian EYHS, 7: HEAPS, 8: IBDS, 9: MAGIC, 10: NHANES 2005-06, 11: Norway EYHS, 12: NHANES 2003-04, 13: PEACH, 14: Pelotas, 15: Portugal EYHS, 16: SPEEDY, 17: TAAG, 18: CHAMPS UK, 19: Ballabeina Study, 20: KISS, 21: CHAMPS US

### Associations between combinations of intensity and bout-duration with cardiometabolic risk factors

**Fig. 2:**
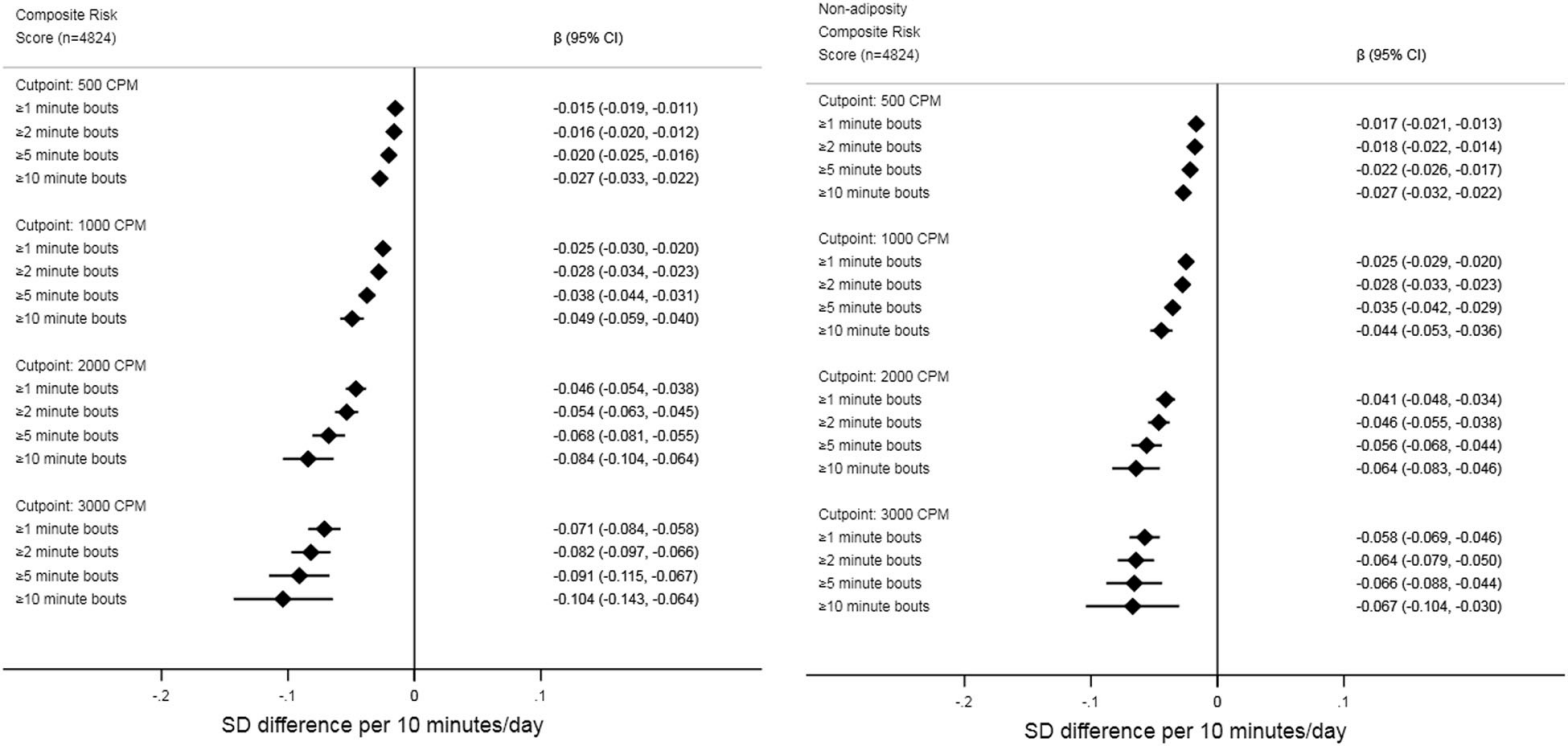
Forest plot of associations between intensity/bout combinations and composite risk scores. Beta-coefficients and 95% CI from linear mixed regression models controlled for age, sex, wear-time including study and participant as “random-effects”. Non-adiposity composite risk score additionally controlled for BMI. Physical activity exposure is based on summarizing all activity exceeding the considered intensity/bout-duration threshold

**Fig. 3:**
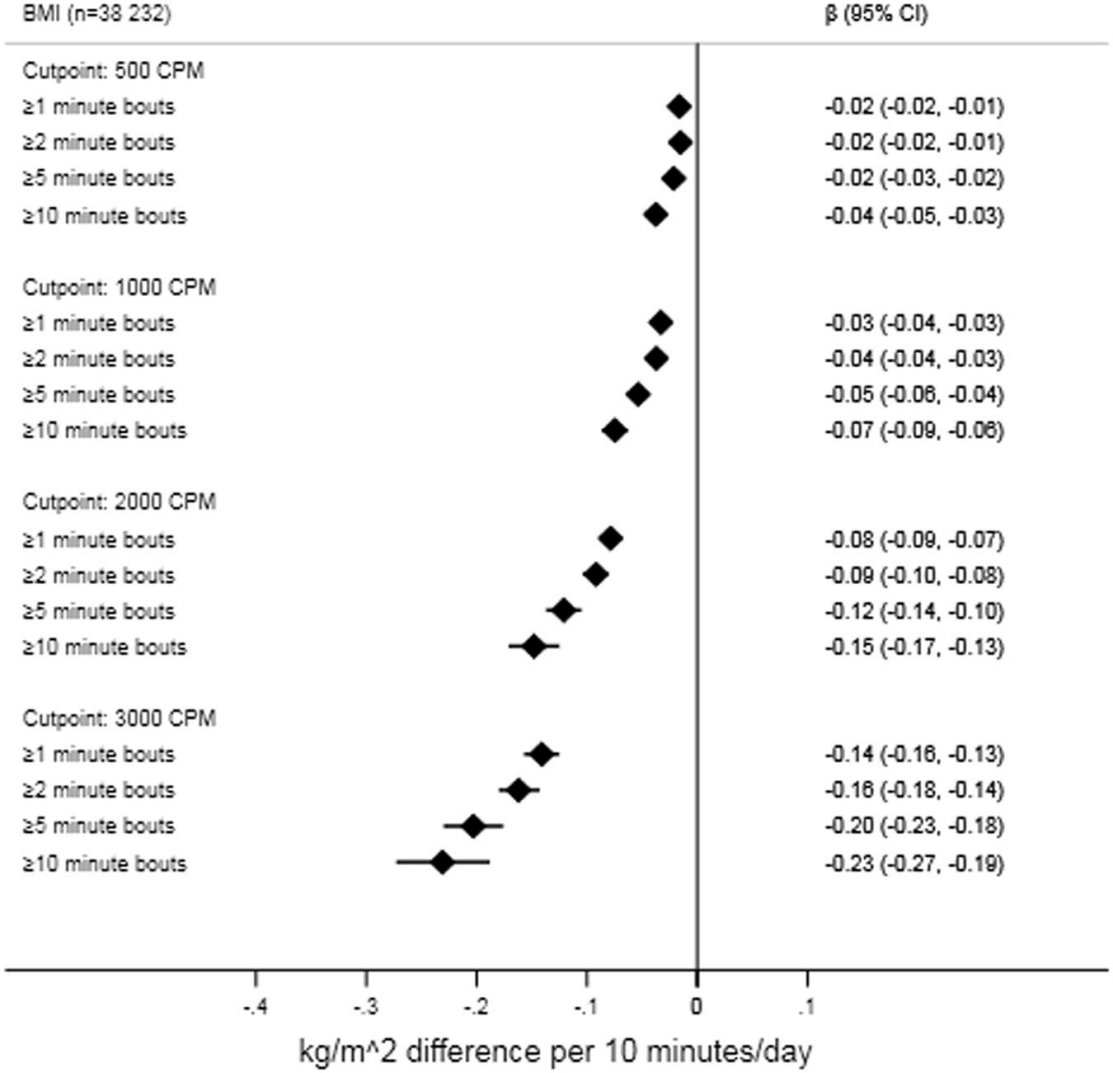
Forest plot of associations between intensity/bout combinations and BMI. Beta-coefficients and 95% CI from linear mixed regression models controlled for age, sex, wear-time including study and participant as “random-effects”. Physical activity exposure is based on summarizing all activity exceeding the considered intensity/bout-duration threshold

Forest plots of bout/intensity combinations and their associations with the composite risk score, non-adiposity composite risk score, and BMI from the linear mixed regression are shown in Figs. 2 and 3. As the overall pattern of association was similar for the remaining outcomes, we show results (with and without BMI adjustment if appropriate) for insulin, glucose, triglyceride, HDL-cholesterol, MAP, waist circumference, and odds of overweight/obesity in Supplementary File Figs S2–S8. Intensity/bout combinations were negatively associated with the cardiometabolic risk factors, suggesting participants with higher activity levels had more favorable risk profiles irrespective of intensity threshold and bout-duration in the range examined. Additional control for BMI attenuated effect-sizes, with attenuation appearing greater (absolute and relative) at higher intensities (Fig. 2 and Supplementary File Figs. S2–S6). Using waist circumference as covariate instead of BMI or exchanging waist circumference for BMI in the composite risk score did not produce noticeable changes (data not shown). Overall, the data suggested a pattern of increasing effect-sizes with activity accumulated at higher intensity thresholds e.g., a 10 min difference in total activity ≥500 cpm was associated with a −0.014 standard deviations (95% CI: −0.018 to −0.01) lower composite risk score and a −0.016 (95% CI: −0.022 to −0.011) kg/m^2^ lower BMI. In comparison, additional 10 min of activity ≥3000 cpm was associated with a −0.069 standard deviations (95%CI: −0.081 to −0.056) lower composite risk score and a −0.141 (95% CI: −0.157 to −0.125) kg/m^2^ lower BMI. A pattern of increasing effect-sizes with increasing bout-durations was observed within all intensity thresholds. For example, a 10 min difference in total activity ≥2000 cpm was associated with a −0.043 standard deviations (95% CI: −0.051 to −0.035) lower composite risk score, while 10 min of the same intensity accrued in medium, and long bouts was associated with a −0.065 (95% CI: −0.078 to −0.052) and −0.081 (95% CI: −0.101 to −0.061) standard deviations lower composite score, respectively. Effect-sizes for glucose and triglycerides followed an irregular pattern at ≥2000 cpm and ≥3000 cpm with weaker associations observed with medium and long bout-durations (Supplementary File Fig. S3 and S4). The mean BMI of the quartile spending the highest percentage of time ≥500 cpm (>32.9% of wear time) was 0.28 (95%CI: 0.19 to 0.36) kg/m^2^ lower than the quartile spending the least time above the threshold (<20.6% of wear time). Being in the most active quartile of physical activity ≥3000 cpm (>4.9% of wear time) was associated with a 0.80 (95%CI: 0.71 to 0.89) kg/m^2^ lower BMI in comparison with the quartile accumulating the least activity above the threshold (<1.8% of wear time). Adding age-by-intensity/bout-duration interaction terms in separate models did not support heterogeneity of associations across participant age for the composite risk score. Conversely, a pattern of negative age-by-intensity/bout-duration interaction terms were observed for BMI, suggesting higher intensities and bout-durations were associated with larger effect-sizes in older participants. Weight status (normal weight versus overweight/obese) modified the associations as indicated by statistically significant weight status-by-intensity/bout-duration interaction terms for both the composite risk score and BMI. The magnitude of associations was stronger in overweight/obese participants than in their normal weight peers, particularly for BMI (Supplementary File Figs. S9 and S10). The pattern of associations did not differ across weight status for the composite risk score.

Meta-regression suggested independent contributions of intensity for all outcomes except for glucose and triglyceride, wherein CI’s overlapped the null (coefficients shown in Supplementary File Table S3). No statistical support for independent effects of bout-duration on outcomes was found. Each 1000 cpm increase in the activity threshold was associated with a −0.026 (−0.039 to −0.014) standard deviations and a −0.064 (−0.09 to −0.038) kg/m^2^ difference in the beta-coefficient for the composite score and BMI, respectively. When adding the intensity-by-bout duration interaction term, this did not reach statistical significance for any outcome (coefficients shown in Supplementary File table S3).

### Isotemporal substitution of short for medium and long bouts of physical activity

Supplementary file Table S4 includes quintiles of residual variation in bouted physical activity after controlling for total activity volume (≥1 min bouts), sex, age, and wear-time. Associations with isotemporal activity substitution and the composite risk score, non-adiposity risk score and BMI are shown in Table 3 (other outcomes shown in Supplementary File Tables S5 and S6). Replacing 10 min/day of activity accumulated in short bouts with an identical amount of same intensity time accumulated in medium or long bouts, produced mixed associations with the cardiometabolic risk factors. E.g., substituting short bout activity above 500 cpm with physical activity accumulated in long bouts was associated with a −0.032 (95% CI: −0.047 to −0.018) standard deviation lower composite score, but substituting 10 min of short bout activity above 3000 cpm with the same amount of activity accumulated in long bouts was associated with a 0.066 (95% CI: 0.013 to 0.118) standard deviation *higher* composite score.

**Table 3:** Associations for composite risk score, non-adiposity risk score, and BMI from isotemporal substitutionof short to medium and long bouts of physical activity

	Composite risk score (*n* = 4338)			Non-adiposity composite risk score (*n* = 4338)^a^			BMI (*n* = 38,232)		
	Beta	95% CI	*p*-value	Beta	95% CI	*p*-value	Beta	95% CI	*p*-value
Medium bouts_500 cpm_	−0.06	−0.035 to 0.025	0.68	−0.02	−0.043 to 0.011	0.25	0.103	0.071 to 0.135	<0.001
Long bouts_500 cpm_	−0.03	−0.047 to −0.018	<0.001	−0.03	−0.040 to −0.013	<0.001	−0.02	−0.034 to 0.0002	0.052
Medium bouts_1000 cpm_	−0.04	−0.075 to −0.003	0.03	−0.04	−0.070 to −0.002	0.04	−0.02	−0.061 to 0.019	0.29
Long bouts_1000 cpm_	−0.04	−0.062 to −0.026	<0.001	−0.03	−0.046 to −0.012	0.001	−0.08	-0.100 to −0.058	<0.001
Medium bouts_2000 cpm_	−0.04	−0.091 to 0.015	0.17	0.057	−0.222 to 0.336	0.69	−0.08	−0.136 to −0.022	0.006
Long bouts_2000 cpm_	−0.01	−0.037 to 0.026	0.72	0.172	0.012 to 0.333	0.04	−0.07	−0.104 to −0.036	<0.001
Medium bouts_3000 cpm_	0.081	0.003 to 0.158	0.04	0.105	0.032 to 0.177	0.005	−0	−0.089 to 0.081	0.92
Long bouts_3000 cpm_	0.066	0.013 to 0.118	0.01	0.099	0.050 to 0.149	<0.001	0.022	−0.034 to 0.079	0.44

^a^Non-adiposity composite risk score controlled for BMI

Beta-coefficients with 95% confidence intervals from linear mixed regression models. Coefficients are interpreted as holding the volume of physical activity above the respective threshold constant, but replacing 10 min of physical activity accumulated in shorter bouts (1-4 min) with 10 min of same intensity physical activity accumulated in the respective bout-duration (≥5–9 (medium) or ≥10 (long) minute bouts). *Cpm* counts/min, *CI* confidence interval, *BMI* body mass index

## Discussion

These data suggest time spent in physical activity with increasing intensity is favorably associated with cardiometabolic risk markers in youth irrespective of bout-duration. Activity accumulated at higher intensities produced progressively greater magnitude of associations as indicated by lower levels of risk markers and a favorable body composition within the range of intensity and bout-duration examined. Meta-regression and isotemporal substitution models provided no evidence for an additional benefit of bouted activity above that of a strong correlation with total physical activity.

### Physical activity intensity

It is well-established that physical activity improves cardiometabolic risk factors in children and adolescents [4, 6, 48]. In epidemiological studies, physical activity is often operationalized by MVPA which is frequently derived by counting time spent at or above 2000 to 3200 cpm in youth [18]. We show that favorable associations with the cardiometabolic risk factors are already present at the lower end of the intensity spectrum, in the range of what is often considered light intensity physical activity, but effect-sizes increase in magnitude as intensity increases. In secondary models including control for adiposity we observed attenuation of effect-sizes. However, consistent with previous reports [49] adding adiposity to models did not fully attenuate the beneficial associations with physical activity. Models stratified by weight status supported this finding. Our operationalization of intensity included all activity above the respective threshold and did not consider an isolated intensity range (e.g., light intensity physical activity, moderate intensity physical activity). As such, our estimates of e.g., ≥500 cpm would include more than one of the “conventional” intensity domains without distinguishing their relative contributions to the estimate. It would therefore be premature to promote physical activities within the lower end of the intensity spectrum for cardiometabolic benefits based on these data. In young people the role of light or total volume of physical activity intensity on the risk markers appears less convincing than that of higher intensity activities [50, 51]. Intensity-dependent associations are consistent with the results of randomized-controlled trials comparing high-intensity interval training with continuous lower intensity exercise [52]. Our data supports the recommendation of high intensity intermittent activity patterns for cardiometabolic benefits in young people. In children, 3000 cpm corresponds to walking at approximately 4–5 km/h [53, 54] and physical activity above this intensity threshold should thus be readily attained by healthy individuals. As the sample spent on average ≈3% of their time above 3000 cpm and less than 50% of boys and girls accumulated any 10-min bout above this intensity threshold, increasing activities at particularly higher intensities may provide guidance for public health actions and interventions. An additional argument for higher intensity activity is potential fitness adaptations as higher fitness-levels in adolescence are strongly and inversely linked with future risk of cardiovascular disease [55].

### Physical activity bouts

Understanding the cardiometabolic benefits associated with physical activity accumulated in intermittent or continuous patterns have significant practical implications. For example, will the cardiometabolic benefits of activity accumulated in short intervals throughout the day be inferior to those of a prolonged continuous session? Achievement of cardiometabolic benefits irrespective of bout-duration would increase feasibility as some may find it more appealing to incorporate shorter activity bouts than having to allocate an extended period of time. It may also be easier to implement short breaks of high intensity activity into the school day than to prioritize resources for a longer continuous session. Consistent with existing literature, a strong correlation between bouted and total time engaged in physical activity was observed [14, 17]. This suggests a direct comparison of cardiometabolic risk factor associations between physical activity accumulated in shorter and longer bout-durations would be confounded by the amount of total activity. Therefore, an isotemporal substitution approach was used to model the impact of replacing short bouts with medium and long bouts of activity while holding total activity constant. These models did not suggest physical activity accumulated in longer bout-durations will produce more pronounced benefits than shorter bouts (at least down to a 1 min bout) when the total volume (time and intensity) of activity are identical. Meta-regression supported this notion as neither the coefficient for bout-duration nor the interaction between bout-duration and intensity was statistically significant. The conclusion of no evidence for an additional benefit of long bouted activity above that of short bouted activity on cardiometabolic risk markers was also reached in a recent systematic review [51]. However, any additional health benefit from longer bouts of activity is difficult to extract from the literature as studies are discordant in their analytical approach for examining this issue. Any specific biological mechanism favouring longer bout-durations under identical total volumes of activity also remain unidentified.

### Limitations

The pattern of results for substitution models was counterintuitive with conflicting directions of associations. This could suggest issues with collinearity which was indeed large. However, residual-analysis indicated meaningful (i.e., reasonable intervention target) variation in bouted activity remained after controlling for total activity and correlations did not differ substantially in magnitude from what is reported from e.g., substitution of distinct fatty acids [56]. Accumulation of particularly longer bouts of activity at higher intensities was low which could also reduce performance of the substitution models because of insufficient information. A min-for-min comparison of short and long bouts above a certain threshold may in fact be confounded by the intensity of the underlying behavior if the contrast is not isocaloric. The direction of this potential bias is likely to inflate effect-sizes for longer bout-durations [57]. Analogously, the substitution models did not account for activity below the intensity threshold, which could also be discordant between individuals engaging in activities of short and long bout-durations. The applied definition of bouts did not allow for interruptions in the time-series, and it remains unclear whether bouted behaviors are better captured by allowing for interruptions. Children’s physical activity is sporadic suggesting bout-durations and time above a given intensity threshold may be misclassified using a 60-s epoch as compared to a shorter epoch-duration. We expect this misclassification to be non-differential in relation to outcomes. Data was cross-sectional so we are unable to infer the direction of association. Prospective studies are needed to establish the temporal nature of our findings. It may be speculated that reverse causation bias is unlikely for cardiometabolic risk factors whereas the association with BMI or waist circumference may be bi-directional [58]. We are also unaware of any controlled studies which have robustly examined the impact of short compared with longer bouts of habitual physical activity on cardiometabolic risk markers. Finally, we only controlled for age, sex and study, hence the possibility of bias owing to uncontrolled confounding sources such as diet quality and quantity [59], socioeconomic possibilities [60], and sexual maturity cannot be rejected. Uncontrolled confounding from these or other sources could significantly influence the strength and pattern of the observed associations.

## Conclusions

In this international observational study including up to 30,000 youth, physical activity intensity appeared a major determinant of variation in cardiometabolic risk factors within the ranges of intensity and bout-duration examined. Greater magnitudes of associations were consistently observed at higher intensities. These results do not support the inclusion of specific bout-durations in youth activity recommendations but suggests that physical activity, preferably at higher intensities, of any accumulation pattern should be promoted by authorities, clinicians, and parents to improve cardiovascular health in young people.

### Availability of data and materials

Data from ICAD is available per request as a supported access resource. Analyzed data is de-identified from the main ICAD database, available only for the approved analyses, and cannot be shared by the authors. Analyses included in this manuscript can be reproduced by requesting a new data release.

## Electronic supplementary material

Supplementary File
