# Correction to: Physical activity intensity, bout-duration, and cardiometabolic risk markers in children and adolescents

**DOI:** 10.1038/s41366-019-0465-2

**Published:** 2019-10-07

**Authors:** Jakob Tarp, Abbey Child, Tom White, Kate Westgate, Anna Bugge, Anders Grøntved, Niels Wedderkopp, Lars B. Andersen, Greet Cardon, Rachel Davey, Kathleen F. Janz, Susi Kriemler, Kate Northstone, Angie S. Page, Jardena J. Puder, John J. Reilly, Luis B. Sardinha, Esther M. F. van Sluijs, Ulf Ekelund, Katrien Wijndaele, Søren Brage, On behalf of the International Children’s Accelerometry Database (ICAD) Collaborators

**Affiliations:** 1 Research Unit for Exercise Epidemiology, Department of Sports Science and Clinical Biomechanics, Centre of Research in Childhood Health, University of Southern Denmark, Odense, Denmark; 2 Medical Research Council Epidemiology Unit, University of Cambridge, Cambridge, UK; 3 University of Cambridge, Cambridge, UK; 4 Sports Medicine Clinic, The Orthopedic Department, Hospital of Lillebaelt Middelfart, Institute of Regional Health Research, University of Southern Denmark, Odense, Denmark; 5 Department of Teacher Education and Sport, Western Norwegian University of Applied Sciences, Sogndal, Norway; 6 Department of Movement and Sports Sciences, Ghent University, 9000 Ghent, Belgium; 7 Centre for Research and Action in Public Health, University of Canberra, Canberra, ACT Australia; 8 Department of Health and Human Physiology, University of Iowa, Iowa City, IA USA; 9 Epidemiology, Biostatistics and Prevention Institute, University of Zurich, Zurich, Switzerland; 10 Bristol Medical School, University of Bristol, Bristol, UK; 11 Centre for Exercise, Nutrition and Health Sciences, School for Policy Studies, University of Bristol, Bristol, UK; 12 Service of Endocrinology, Diabetes and Metabolism and Division of Pediatric Endocrinology, Diabetes and Obesity, University Hospital Lausanne, Lausanne, Switzerland; 13 University of Strathclyde, Physical Activity for Health Group, School of Psychological Sciences and Health, Glasgow, Scotland, UK; 14 Exercise and Health Laboratory, Faculty of Human Kinetics, Universidade de Lisboa, Lisbon, Portugal; 15 Centre for Diet and Activity Research (CEDAR), University of Cambridge, Cambridge, UK; 16 Department of Sports Medicine, Norwegian School of Sport Sciences, Oslo, Norway

**Keywords:** Risk factors, Epidemiology, Paediatrics, Lifestyle modification

**Correction to: International Journal of Obesity**

10.1038/s41366-018-0152-8

In the original article, four authors (Dr AJ Atkin, Dr DW Eslinger, Dr BH Hansen and Dr LB Sherar) were not included in the affiliations. This has been corrected in the XML, PDF and HTML versions of this article.

