# Supplementary material for: Physical activity intensity, bout-duration, and cardiometabolic risk markers in children and adolescents
Source: Int J Obes (Lond). 2018 Jul 13;42(9):1639–50. doi: 10.1038/s41366-018-0152-8 (PMC6160399; doi:10.1038/s41366-018-0152-8)
Supplement: Supplementary file 1 — Supplementary File [file 41366_2018_152_MOESM1_ESM.docx]

Supplementary File

Jakob Tarp, Abbey Child, Tom White, Kate Westgate, Anna Bugge, Anders Grøntved, Niels Wedderkopp, Lars B Andersen, Greet Cardon, Rachel Davey, Kathleen F Janz, Susi Kriemler, Kate Northstone, Angie S. Page, Jardena J. Puder, John J Reilly, Luis B Sardinha, Esther MF van Sluijs, Ulf Ekelund, Katrien Wijndaele and Søren Brage On behalf of the International Children’s Accelerometry Database (ICAD) Collaborators

Table of contents

Page 3: Table S1. Study characteristics

Page 4: Figure S1 Age distribution of included participants

Page 5: Table S2. Correlation matrix of physical activity intensity/bout-duration combinations

Page 6: Figure S2. Forest plot of associations between intensity/bout combinations and insulin levels

Page 7: Figure S3. Forest plot of associations between intensity/bout combinations and glucose levels

Page 9: Figure S4. Forest plot of associations between intensity/bout combinations and triglyceride levels

Page 9: Figure S5. Forest plot of associations between intensity/bout combinations and mean arterial pressure

Page 10: Figure S6. Forest plot of associations between intensity/bout combinations and HDL-cholesterol

Page 11: Figure S7. Forest plot of associations between intensity/bout combinations and waist-circumference

Page 12: Figure S8. Forest plot of intensity/bout combinations and odds of overweight/obesity

Page 13: Figure S9. Forest plots of associations between intensity/bout combinations and composite risk score stratified by weight status

Page 14: Figure S10. Forest plots of associations between intensity/bout combinations and BMI stratified by weight status

Page 15: Table S3. Meta-regression coefficients

Page 16: Table S4. Quintiles of bouted physical activity after accounting for variation due to differences in total physical activity

Page 17: Table S5. Isotemporal substitution of non-bout to ≥5-9 and ≥10 minutes physical activity associations with insulin, glucose, and triglyceride

Page 18: Table S6. Isotemporal substitution of non-bout to ≥5-9 and ≥10 minutes physical activity associations with mean arterial pressure, HDL-cholesterol, and waist-circumference

Table S1. Study characteristics

| Study | Year | N* | Country | Age in years  (median (range))** | Counts/min  (median (IQR))** | Prospective  data included |
| --- | --- | --- | --- | --- | --- | --- |
| ALSPAC | 03-07 | 6418 | England | 12 (11 - 15) | 549 (446 - 678) | Yes |
| Belgium Pre-School Study | 06; 08-09 | 115 | Belgium | 5 (4 - 6) | 565 (486 - 683) | No |
| CLAN | 01; 04; 06 | 1104 | Australia | 11 (5 - 17) | 662 (538 - 794) | Yes |
| CoSCIS | 01-05 | 607 | Denmark | 7 (6 - 11) | 683 (568 - 808) | Yes |
| Danish EYHS | 97-98; 03-04 | 1193 | Denmark | 10 (8 - 17) | 533 (375 - 699) | Yes |
| Estonian EYHS | 98-99 | 647 | Estonia | 10 (8 - 17) | 595 (432 - 762) | No |
| HEAPS | 02-03; 06 | 1265 | Australia | 11 (4 - 15) | 663 (539 - 810) | Yes |
| IBDS | 98-07 | 576 | US | 6 (5 - 14) | 684 (576 - 793) | Yes |
| MAGIC | 02 | 278 | Scotland | 4 (4 – 5) | 712 (613 - 847) | No |
| NHANES 2005-06 | 05-06 | 2132 | US | 13 (6 - 18) | 471 (348 - 630) | No |
| Norway EYHS | 99-00 | 366 | Norway | 10 (9 - 10) | 688 (546 - 864) | No |
| NHANES 2003-04 | 03-04 | 2046 | US | 13 (6 - 18) | 491 (362 - 650) | No |
| PEACH | 06-09 | 1197 | England | 11 (10 - 13) | 515 (430 - 629) | Yes |
| Pelotas | 06-07 | 270 | Brazil | 13 (13 - 14) | 376 (288 - 477) | No |
| Portugal EYHS | 99-00 | 1050 | Portugal | 10 (8 - 18) | 524 (410 - 664) | Yes |
| SPEEDY | 07 | 1890 | England | 10 (10 - 11) | 563 (460 - 685) | No |
| Project TAGG | 02-06 | 6884 | US | 14 (10 - 17) | 367 (298 - 450) | Yes |
| CHAMPS UK | 06-07 | 454 | England | 11 (4 - 16) | 520 (407 - 644) | No |
| Ballabeina Study | 08-09 | 546 | Switzerland | 5 (4 - 7) | 678 (566 - 793) | Yes |
| KISS | 05-06 | 488 | Switzerland | 10 (6 - 13) | 639 (526 - 773) | Yes |
| CHAMPS-US | 03-06 | 274 | US | 5 (4 - 6) | 701 (592 - 795) | No |

*Unique individual participants. **Only observations available at "baseline" included if prospective data available

Figure S1. Age distribution of included participants (n=38 306).

Table S2. Correlation matrix of physical activity intensity/bout-duration combinations

|  | 500  cpm  1-min | 500  cpm  2-min | 500  cpm  5-min | 500  cpm  10-min | 1000  cpm  1-min | 1000  cpm  2-min | 1000  cpm  5-min | 1000  cpm  10-min | 2000  cpm  1-min | 2000  cpm  2-min | 2000  cpm  5-min | 2000  cpm  10-min | 3000  cpm  1-min | 3000  cpm  2-min | 3000  cpm  5-min | 3000  cpm  10-min |
| --- | --- | --- | --- | --- | --- | --- | --- | --- | --- | --- | --- | --- | --- | --- | --- | --- |
| 500cpm  1-min | - | 0.99 | 0.93 | 0.80 | 0.95 | 0.90 | 0.77 | 0.61 | 0.77 | 0.69 | 0.54 | 0.39 | 0.62 | 0.53 | 0.39 | 0.25 |
| 500cpm  2-min | - | - | 0.96 | 0.84 | 0.96 | 0.93 | 0.81 | 0.65 | 0.80 | 0.72 | 0.57 | 0.42 | 0.65 | 0.56 | 0.41 | 0.27 |
| 500cpm  5-min | - | - | - | 0.93 | 0.96 | 0.96 | 0.91 | 0.76 | 0.85 | 0.80 | 0.67 | 0.50 | 0.71 | 0.64 | 0.49 | 0.32 |
| 500cpm  10-min | - | - | - | - | 0.88 | 0.91 | 0.95 | 0.88 | 0.85 | 0.84 | 0.76 | 0.62 | 0.73 | 0.69 | 0.56 | 0.39 |
| 1000cpm  1-min | - | - | - | - | - | 0.99 | 0.90 | 0.75 | 0.91 | 0.84 | 0.68 | 0.52 | 0.77 | 0.68 | 0.51 | 0.34 |
| 1000cpm  2-min | - | - | - | - | - | - | 0.94 | 0.80 | 0.94 | 0.89 | 0.74 | 0.56 | 0.80 | 0.72 | 0.55 | 0.37 |
| 1000cpm  5-min | - | - | - | - | - | - | - | 0.92 | 0.93 | 0.93 | 0.85 | 0.68 | 0.83 | 0.79 | 0.64 | 0.44 |
| 1000cpm  10-min | - | - | - | - | - | - | - | - | 0.85 | 0.88 | 0.89 | 0.79 | 0.79 | 0.78 | 0.70 | 0.53 |
| 2000cpm  1-min | - | - | - | - | - | - | - | - | - | 0.98 | 0.87 | 0.70 | 0.94 | 0.88 | 0.71 | 0.50 |
| 2000cpm  2-min | - | - | - | - | - | - | - | - | - | - | 0.92 | 0.76 | 0.95 | 0.92 | 0.76 | 0.54 |
| 2000cpm  5-min | - | - | - | - | - | - | - | - | - | - | - | 0.88 | 0.89 | 0.92 | 0.86 | 0.64 |
| 2000cpm  10-min | - | - | - | - | - | - | - | - | - | - | - | - | 0.76 | 0.81 | 0.85 | 0.77 |
| 3000cpm  1-min | - | - | - | - | - | - | - | - | - | - | - | - | - | 0.98 | 0.84 | 0.62 |
| 3000cpm  2-min | - | - | - | - | - | - | - | - | - | - | - | - | - | - | 0.91 | 0.69 |
| 3000cpm  5-min | - | - | - | - | - | - | - | - | - | - | - | - | - | - | - | 0.82 |
| 3000cpm  10-min |  |  |  |  |  |  |  |  |  |  |  |  |  |  |  | - |

Correlations are Spearman’s rho controlling for age, sex, wear-time and study

Figure S2. Forest plot of associations between intensity/bout combinations and insulin levels


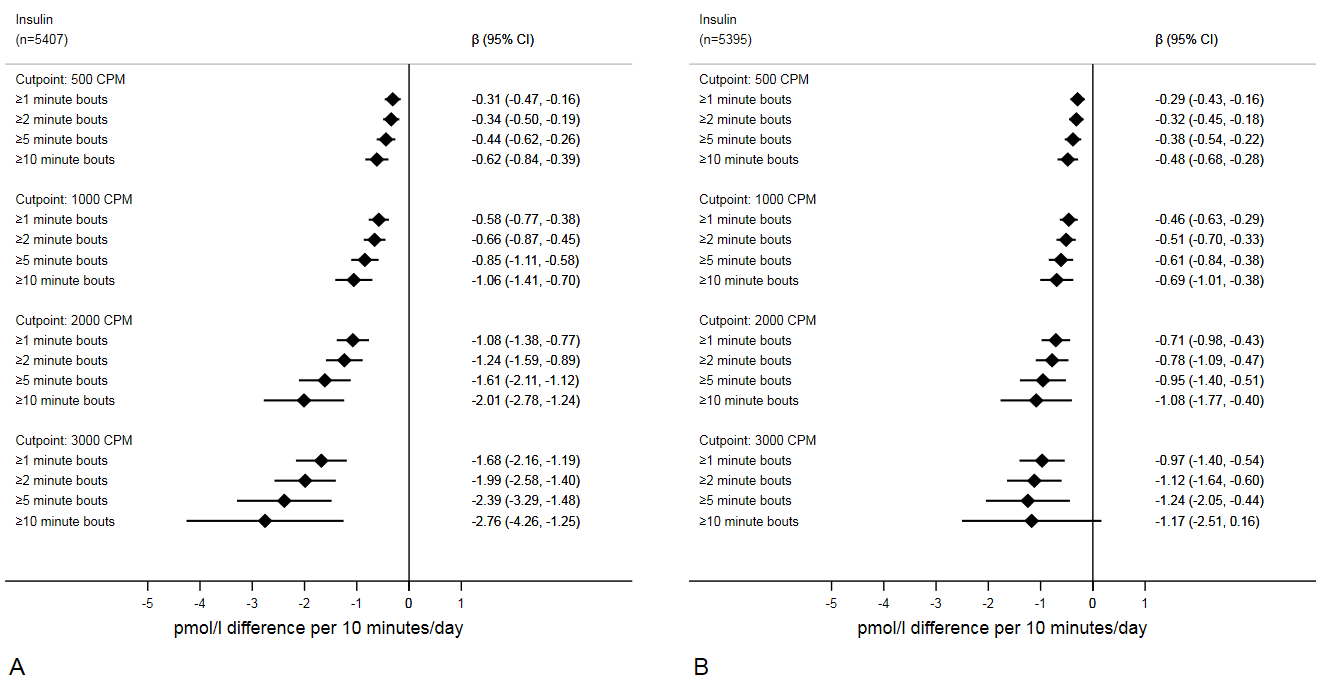


Beta-coefficients and 95% CI from linear mixed regression models controlled for age, sex, wear-time including study and participant as “random-effects” (A). Panel (B) including control for BMI. Physical activity exposure is based on summarizing all activity exceeding the considered intensity/bout-duration threshold.

Figure S3. Forest plot of associations between intensity/bout combinations and glucose levels


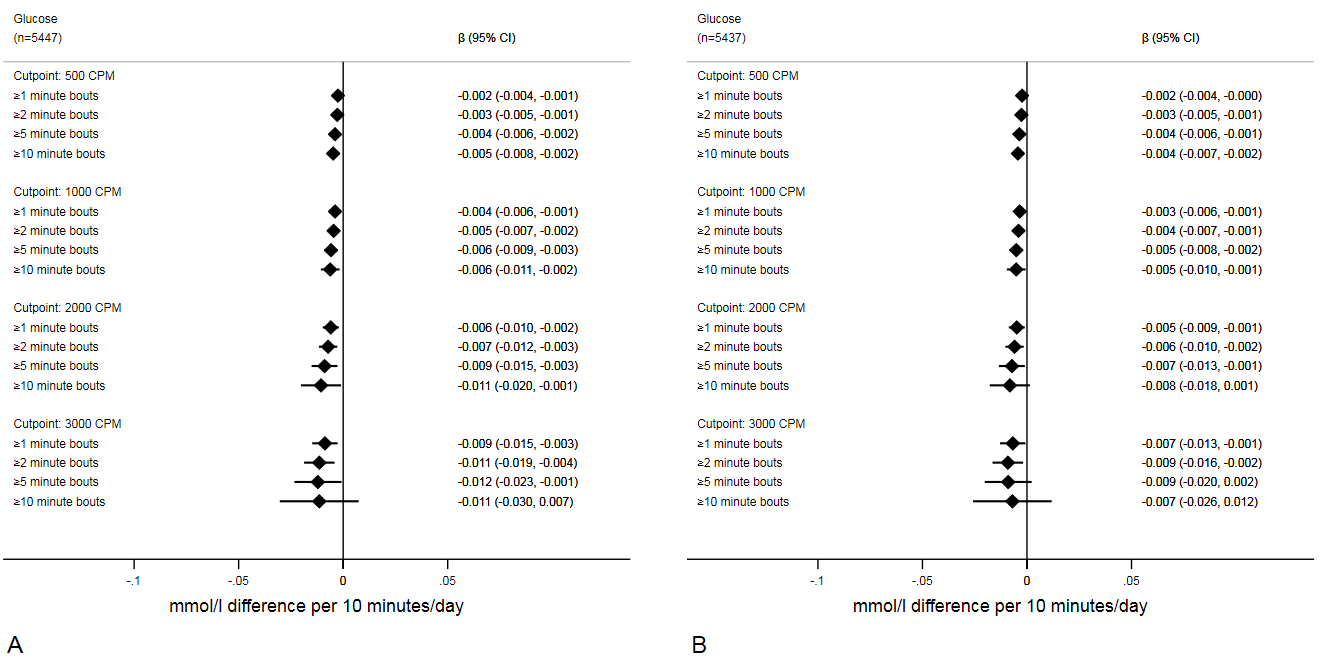


Beta-coefficients and 95% CI from linear mixed regression models controlled for age, sex, wear-time including study and participant as “random-effects” (A). Panel (B) including control for BMI. Physical activity exposure is based on summarizing all activity exceeding the considered intensity/bout-duration threshold

Figure S4. Forest plot of associations between intensity/bout combinations and triglyceride levels


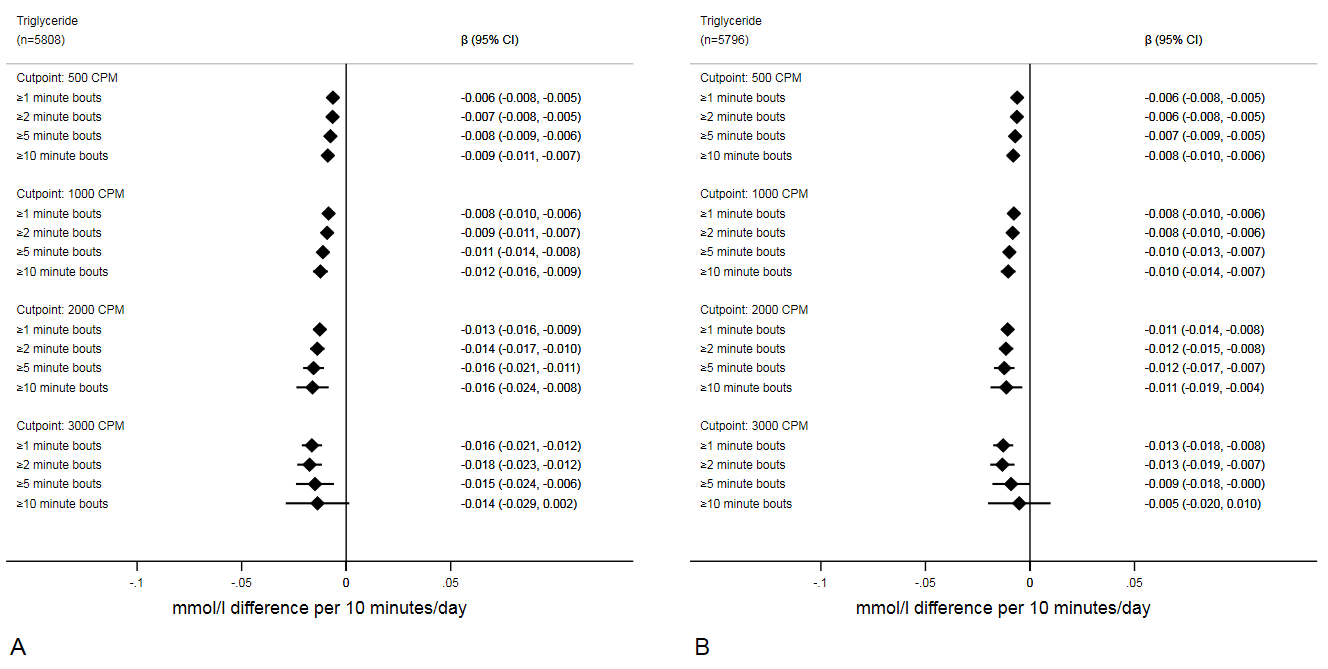


Beta-coefficients and 95 %CI from linear mixed regression models controlled for age, sex, wear-time including study and participant as “random-effects” (A). Panel (B) including control for BMI. Physical activity exposure is based on summarizing all activity exceeding the considered intensity/bout-duration threshold.

Figure S5. Forest plot of associations between intensity/bout combinations and mean arterial pressure


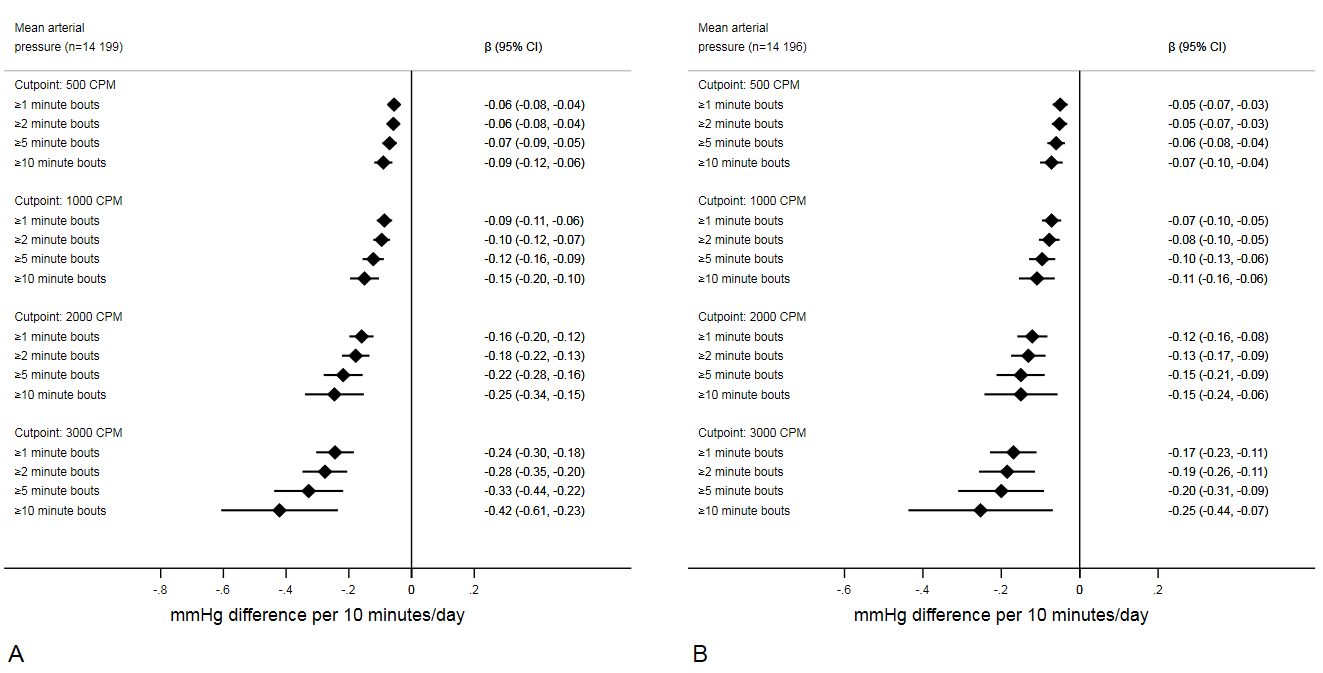


Beta-coefficients and 95% CI from linear mixed regression models controlled for age, sex, body height, wear-time including study and participant as “random-effects” (A). Panel (B) including control for BMI. Physical activity exposure is based on summarizing all activity exceeding the considered intensity/bout-duration threshold.

Figure S6. Forest plot of associations between intensity/bout combinations and HDL-cholesterol


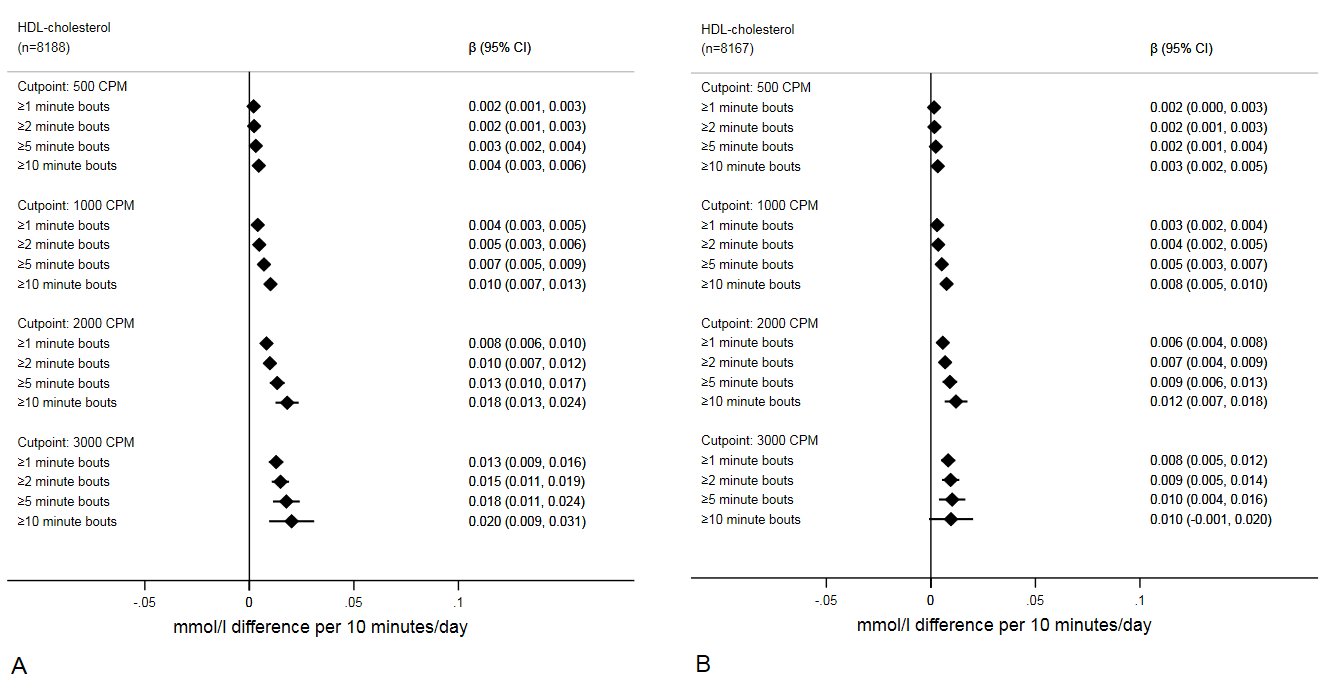


Beta-coefficients and 95% CI from linear mixed regression models controlled for age, sex, wear-time including study and participant as “random-effects” (A). Panel (B) including control for BMI. Physical activity exposure is based on summarizing all activity exceeding the considered intensity/bout-duration threshold.

Figure S7. Forest plot of associations between intensity/bout combinations and waist circumference


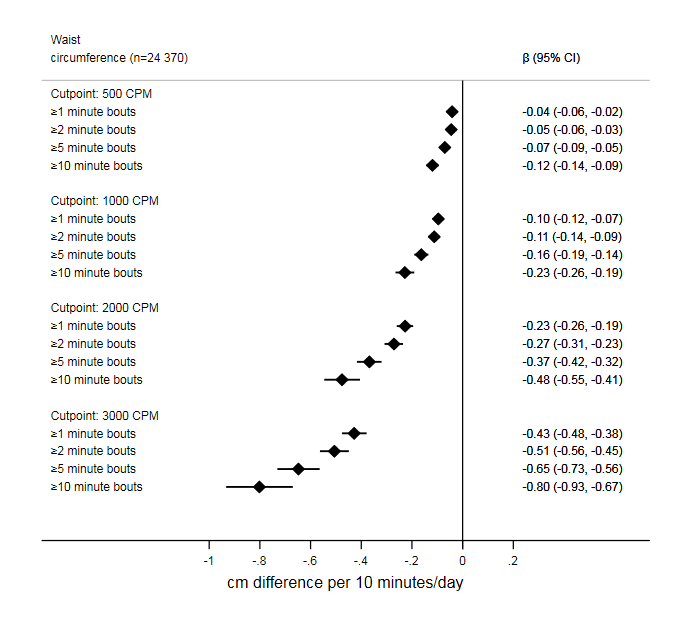


Beta-coefficients and 95% CI from linear mixed regression models controlled for age, sex, body height, wear-time including study and participant as “random-effects”. Waist circumference data harmonized by correction formula. Physical activity exposure is based on summarizing all activity exceeding the considered intensity/bout-duration threshold.

Figure S8. Forest plot of intensity/bout combinations and odds of overweight/obesity


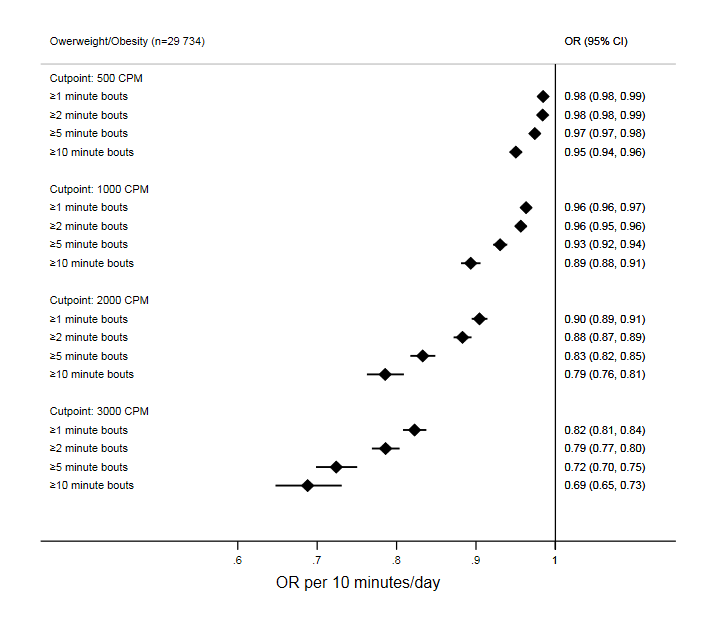


Odds ratio and 95% CI from logistic mixed regression models controlled for age, sex, wear-time and including study as a “random-effects”. Reference is BMI below overweight/obesity according to World Obesity Federation cut-offs^1^. Number of participants included in analysis is lower than for BMI because of non-convergence of mixed models including random intercepts at both the study and participant level. The earliest observation is included when >1 measurement on the same individual was available. Physical activity exposure is based on summarizing all activity exceeding the considered intensity/bout-duration threshold.

Figure S9. Forest plots of associations between intensity/bout combinations and composite risk score stratified by weight status


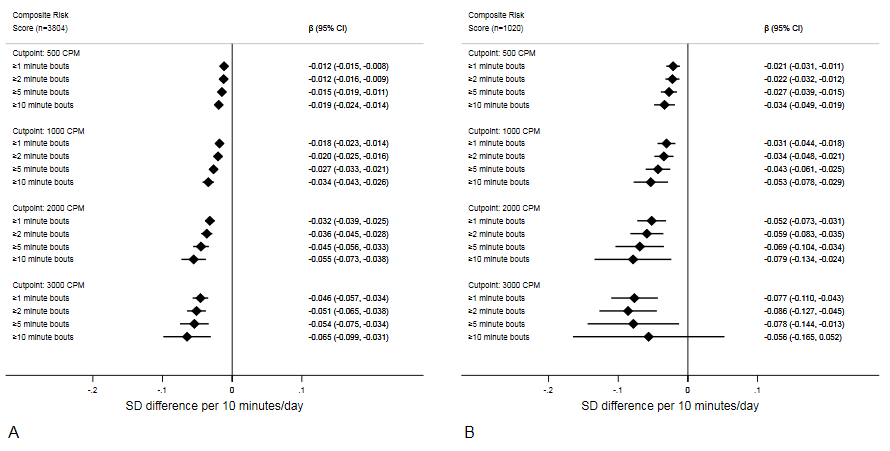


Beta-coefficients and 95% CI from linear mixed regression models controlled for age, sex, wear-time including study and participant as “random-effects” (A; normal weight, B; overweight/obese). Physical activity exposure is based on summarizing all activity exceeding the considered intensity/bout-duration threshold.

Figure S10. Forest plots of associations between intensity/bout combinations and BMI stratified by weight status


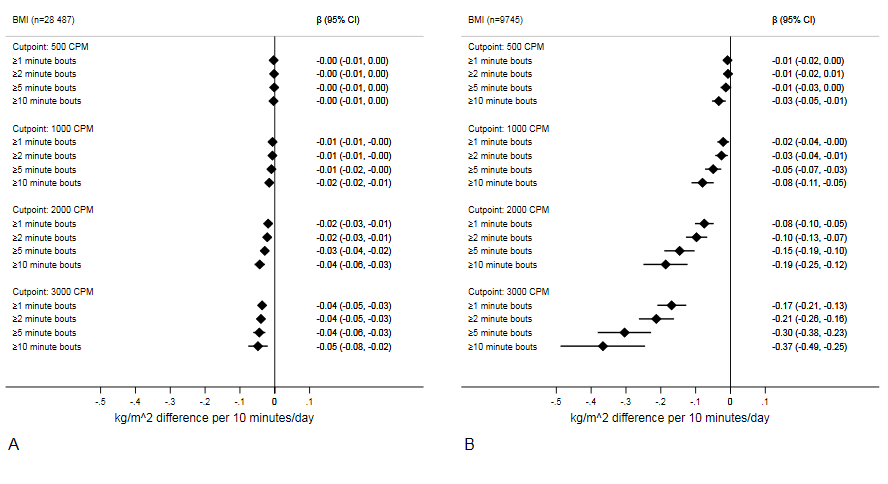


Beta-coefficients and 95% CI from linear mixed regression models controlled for age, sex, wear-time including study and participant as “random-effects” (A; normal weight, B; overweight/obese). Physical activity exposure is based on summarizing all activity exceeding the considered intensity/bout-duration threshold.

Table S3. Meta-regression coefficients

|  | β Bout-  duration  (95% CI) | β Intensity  (95% CI) | Bout-duration-by-intensity  threshold interaction term (95% CI)* |
| --- | --- | --- | --- |
| Composite risk score (z-score) | -0.002 (-0.005 to 0.0005) | -0.027 (-0.039 to -0.014) | -0.002 (-0.006 to 0.003) |
| Non-adiposity composite risk score (z-score) | -0.002 (-0.004 to 0.0008) | -0.019 (-0.031 to -0.007) | -0.0009 (-0.005 to 0.003) |
| Insulin (pmol/l) | -0.048 (-0.138 to 0.042) | -0.667 (-1.075 to -0.259) | -0.048 (-0.222 to 0.126) |
| Glucose (mmol/l) | -0.0003 (-0.002 to 0.0009) | -0.003 (-0.009 to 0.003) | -0.0002 (-0.002 to 0.002) |
| Triglyceride (mmol/l) | -0.0003 (-0.001 to 0.0006) | -0.004 (-0.009 to 0.0005) | -0.00002 (-0.002 to 0.002) |
| HDL-cholesterol (mmol/l) | 0.0005 (-0.0002 to 0.001) | 0.006 (0.002 to 0.009) | 0.0005 (-0.0008 to 0.002) |
| Mean arterial pressure (mmHg) | -0.006 (-0.019 to 0.006) | -0.073 (-0.149 to -0.029) | -0.006 (-0.028 to 0.016) |
| Body mass index (kg/m^2^) | -0.005 (-0.012 to 0.002) | -0.064 (-0.0893 to -0.038) | -0.004 (-0.009 to 0.002) |
| Waist circumference (cm) | -0.019 (-0.048 to 0.01) | -0.208 (-0.31 to -0.11) | -0.013 (-0.033 to 0.006) |

Coefficients per 1-minute increase in bout-duration and 1000 cpm increase in intensity threshold. Meta-regression included estimates from: 1, 3, 5, 7, and 10 minute bout durations and 500, 1000, 2000, and 3000 counts/min intensity thresholds. Confidence intervals adapted to non-independence of coefficients by recalculating the standard error (SE) as: (√(number of coefficients (20) – 1)) x SE obtained from the meta-regression model. *Interaction-term fitted in separate model.

Table S4. Quintiles of bouted physical activity after accounting for variation due to differences in total physical activity (n=38 306)

|  | Quintile 1  (least time in bouts) | Quintile 2 | Quintile 3 | Quintile 4 | Quintile 5 |
| --- | --- | --- | --- | --- | --- |
| Medium bouts_500cpm_ | -21.6 (7.6) | -8.2 (2.5) | -0.3 (2.2) | 7.7 (2.6) | 22.3 (9.1) |
| Long bouts_500cpm_ | -27.0 (9.1) | -10.6 (3.1) | -0.8 (2.7) | 9.3 (3.3) | 29.0 (13.4) |
|  |  |  |  |  |  |
| Medium bouts_1000cpm_ | -17.6 (6.5) | -6.5 (2.0) | -0.2 (1.7) | 6.3 (2.1) | 18.1 (7.6) |
| Long bouts_1000cpm_ | -18.7 (6.8) | -7.2 (2.1) | -0.7 (1.7) | 5.9 (2.2) | 20.7 (10.7) |
|  |  |  |  |  |  |
| Medium bouts_2000cpm_ | -10.8 (4. 3) | -3.8 (1.2) | -0.0 (1.0) | 3.6 (1.2) | 11.1 (5.4) |
| Long bouts_2000cpm_ | -9.6 (3.6) | -3.7 (1.0) | -0.5 (0.8) | 2.7 (1.1) | 11.1 (7.1) |
|  |  |  |  |  |  |
| Medium bouts_3000cpm_ | -6.4 (2.8) | -2.1 (0.7) | -0.1 (0.5) | 1.9 (0.6) | 6.7 (4.3) |
| Long bouts_3000cpm_ | -4.9 (2.0) | -1.8 (0.5) | -0.3 (0.4) | 1.1 (0.5) | 6.0 (5.2) |

Quintiles of residual variation (minutes of activity) from regressing time accumulated in the respective bout-definition on age, sex, wear-time and total physical activity at the intensity threshold. Data is interpreted as obtaining less or more minutes of activity in bouts than expected by the total activity accumulated. Data is mean (SD). Medium (≥5-9 minutes) and long (≥10 minutes).

Table S5. Associations for insulin, glucose, and triglyceride from isotemporal substitution of short to medium and long bouts of physical activity

|  | Insulin (n= 5407) | | | Glucose (n= 5447) | | | Triglyceride (n=5808) | | |
| --- | --- | --- | --- | --- | --- | --- | --- | --- | --- |
|  | Beta | 95% CI | p-value | Beta | 95% CI | p-value | Beta | 95% CI | p-value |
| Medium bouts_500cpm_ | 0.130 | -0.969 to 1.230 | 0.82 | -0.017 | -0.031 to -0.003 | 0.02 | -0.006 | -0.017 to 0.006 | 0.322 |
| Long bouts_500cpm_ | -0.765 | -1.315 to -0.215 | 0.006 | -0.011 | -0.019 to -0.005 | 0.001 | -0.005 | -0.011 to 0.0002 | 0.061 |
|  |  |  |  |  |  |  |  |  |  |
| Medium bouts_1000cpm_ | -0.958 | -2.320 to 0.403 | 0.17 | -0.021 | -0.039 to -0.004 | 0.02 | -0.016 | -0.030 to -0.003 | 0.02 |
| Long bouts_1000cpm_ | -0.820 | -1.499 to -0.141 | 0.02 | -0.006 | -0.015 to 0.002 | 0.14 | -0.004 | -0.011 to 0.003 | 0.272 |
|  |  |  |  |  |  |  |  |  |  |
| Medium bouts_2000cpm_ | -0.884 | -2.856 to 1.087 | 0.38 | -0.008 | -0.034 to 0.017 | 0.53 | 0.001 | -0.019 to 0.021 | 0.937 |
| Long bouts_2000cpm_ | -0.156 | -1.362 to 1.050 | 0.80 | 0.000 | -0.015 to 0.015 | 0.98 | 0.017 | 0.005 to 0.029 | 0.006 |
|  |  |  |  |  |  |  |  |  |  |
| Medium bouts_3000cpm_ | 1.037 | -1.895 to 3.969 | 0.49 | -0.002 | -0.039 to 0.036 | 0.92 | 0.054 | 0.025 to 0.084 | <0.001 |
| Long bouts_3000cpm_ | 1.265 | -0.757 to 3.286 | 0.22 | 0.012 | -0.014 to 0.037 | 0.37 | 0.041 | 0.021 to 0.062 | <0.001 |

Beta-coefficients with 95% confidence intervals from linear mixed regression models. Coefficients are interpreted as holding the volume of physical activity above the respective threshold constant, but replacing 10 minutes of physical activity accumulated in shorter bouts (1-4 minutes) with 10 minutes of same intensity physical activity but accumulated in the respective bout-duration (≥5-9 (medium) or ≥10 (long) minute bouts). Cpm: counts/min, CI: confidence interval.

Table S6. Associations for mean arterial pressure, HDL-cholesterol, and waist-circumference from isotemporal substitution of short to medium and long bouts of physical activity

|  | Mean arterial pressure (n= 14 199) | | | HDL-cholesterol (n= 8188) | | | Waist-circumference (n=24 370 )* | | |
| --- | --- | --- | --- | --- | --- | --- | --- | --- | --- |
|  | Beta | 95% CI | p-value | Beta | 95% CI | p-value | Beta | 95% CI | p-value |
| Medium bouts_500cpm_ | -0.005 | -0.147 to 0.137 | 0.95 | 0.001 | -0.007 to 0.009 | 0.720 | 0.136 | 0.029 to 0.244 | 0.013 |
| Long bouts_500cpm_ | -0.071 | -0.142 to -0.0004 | 0.049 | 0.007 | 0.003 to 0.011 | <0.001 | -0.164 | -0.220 to -0.108 | <0.001 |
|  |  |  |  |  |  |  |  |  |  |
| Medium bouts_1000cpm_ | -0.120 | -0.294 to 0.055 | 0.18 | 0.007 | -0.002 to 0.017 | 0.142 | -0.146 | -0.279 to -0.013 | 0.031 |
| Long bouts_1000cpm_ | -0.091 | -0.176 to -0.005 | 0.04 | 0.013 | 0.008 to 0.017 | <0.001 | -0.287 | -0.355 to -0.219 | <0.001 |
|  |  |  |  |  |  |  |  |  |  |
| Medium bouts_2000cpm_ | 0.086 | -0.338 to 0.165 | 0.50 | 0.007 | -0.008 to 0.021 | 0.358 | -0.271 | -0.456 to -0.085 | 0.004 |
| Long bouts_2000cpm_ | 0.070 | -0.074 to 0.215 | 0.34 | 0.007 | -0.001 to 0.016 | 0.085 | -0.302 | -0.410 to -0.193 | <0.001 |
|  |  |  |  |  |  |  |  |  |  |
| Medium bouts_3000cpm_ | 0.379 | 0.008 to 0.749 | 0.045 | -0.008 | -0.030 to 0.013 | 0.445 | -0.020 | -0.293 to 0.254 | 0.889 |
| Long bouts_3000cpm_ | 0.196 | -0.057 to 0.449 | 0.13 | -0.012 | -0.026 to 0.003 | 0.117 | -0.079 | -0.258 to 0.099 | 0.384 |

Beta-coefficients with 95% confidence intervals from linear mixed regression models. Coefficients are interpreted as holding the volume of physical activity above the respective threshold constant, but replacing 10 minutes of physical activity accumulated in shorter bouts (1-4 minutes) with 10 minutes of same intensity physical activity but accumulated in the respective bout-duration (≥5-9 (medium) or ≥10 (long) minute bouts). Cpm: counts/min, CI: confidence interval. *Waist-circumference harmonized by correction formula.

1. Cole TJ, Bellizzi MC, Flegal KM, Dietz WH. Establishing a standard definition for child overweight and obesity worldwide: international survey. *BMJ* 2000; **320**(7244)**:** 1240-3.
